# Minimal Residual Disease Prior to and After Haematopoietic Stem Cell Transplantation in Children and Adolescents With Acute Lymphoblastic Leukaemia: What Level of Negativity Is Relevant?

**DOI:** 10.3389/fped.2021.777108

**Published:** 2021-11-05

**Authors:** Pietro Merli, Marianne Ifversen, Tony H. Truong, Hanne V. Marquart, Jochen Buechner, Matthias Wölfl, Peter Bader

**Affiliations:** ^1^Department of Pediatric Hematology/Oncology, Cell and Gene Therapy, Bambino Gesù Children's Hospital, IRCCS, Rome, Italy; ^2^Pediatric Stem Cell Transplant and Immune Deficiency, Department of Paediatrics and Adolescent Medicine, Copenhagen University Hospital Rigshospitalet, Copenhagen, Denmark; ^3^Division of Pediatric Oncology and Bone Marrow Transplant, Alberta Children's Hospital, University of Calgary, Calgary, AB, Canada; ^4^Section for Diagnostic Immunology, Department of Clinical Immunology, Copenhagen University Hospital, Copenhagen, Denmark; ^5^Department of Pediatric Hematology and Oncology, Oslo University Hospital, Oslo, Norway; ^6^Pediatric Hematology, Oncology and Stem Cell Transplantation, Children's Hospital, Würzburg University Hospital, Würzburg, Germany; ^7^Division for Stem Cell Transplantation, Immunology and Intensive Care Medicine, Department for Children and Adolescents, Goethe University, University Hospital Frankfurt, Frankfurt, Germany

**Keywords:** minimal residual disease (MRD), acute lymphoblastic leukaemia (ALL), haematopoietic stem cell transplantation (HSCT), children, relapse

## Abstract

Minimal residual disease (MRD) assessment plays a central role in risk stratification and treatment guidance in paediatric patients with acute lymphoblastic leukaemia (ALL). As such, MRD prior to haematopoietic stem cell transplantation (HSCT) is a major factor that is independently correlated with outcome. High burden of MRD is negatively correlated with post-transplant survival, as both the risk of leukaemia recurrence and non-relapse mortality increase with greater levels of MRD. Despite growing evidence supporting these findings, controversies still exist. In particular, it is still not clear whether multiparameter flow cytometry and real-time quantitative polymerase chain reaction, which is used to recognise immunoglobulin and T-cell receptor gene rearrangements, can be employed interchangeably. Moreover, the higher sensitivity in MRD quantification offered by next-generation sequencing techniques may further refine the ability to stratify transplant-associated risks. While MRD quantification from bone marrow prior to HSCT remains the state of the art, heavily pre-treated patients may benefit from additional staging, such as using ^18^F-fluorodeoxyglucose positron emission tomography/computed tomography to detect focal residues of disease. Additionally, the timing of MRD detection (i.e., immediately before administration of the conditioning regimen or weeks before) is a matter of debate. Pre-transplant MRD negativity has previously been associated with superior outcomes; however, in the recent For Omitting Radiation Under Majority age (FORUM) study, pre-HSCT MRD positivity was associated with neither relapse risk nor survival. In this review, we discuss the level of MRD that may require pre-transplant therapy intensification, risking time delay and complications (as well as losing the window for HSCT if disease progression occurs), as opposed to an adapted post-transplant strategy to achieve long-term remission. Indeed, MRD monitoring may be a valuable tool to guide individualised treatment decisions, including tapering of immunosuppression, cellular therapies (such as donor lymphocyte infusions) or additional immunotherapy (such as bispecific T-cell engagers or chimeric antigen receptor T-cell therapy).

## Introduction

During the last decades, minimal (or measurable) residual disease (MRD) quantification has been proven as the leading assessment tool in the evaluation of treatment response and stratification of patient risk in acute lymphoblastic leukaemia (ALL) (1–6). Stratification based on MRD is now incorporated in almost all international protocols for front-line ALL treatment and the management of first relapse. Moreover, persistent or recurring positive MRD is one of the main indications for proceeding to allogeneic haematopoietic stem cell transplantation (HSCT), which is used as consolidation therapy in patients at high risk of relapse (7).

Noticeably, a recent study combining the results of 39 trials conducted in paediatric patients and adults using either multiparametric flow cytometry (MFC) or polymerase chain reaction (PCR)-based approaches to quantify MRD showed that persistence of MRD in non-HSCT trials was consistently associated with inferior prognosis regardless of trial approach and method of MRD detection (8). This finding highlights a need for interventions in MRD-positive patients and also suggests that MRD response could be used as an early endpoint to assess the effectiveness of different therapies.

Similarly to non-HSCT studies, evidence has been accumulated regarding the usefulness of MRD measurement immediately before and after HSCT for defining the risk of relapse and transplant-related mortality (TRM). Thus, MRD assessment may allow the adoption of personalised HSCT approaches (e.g., rapid tapering of post-HSCT immunosuppression in patients at high risk of relapse).

In this review, we focus on the prognostic role of MRD measurement in the setting of HSCT, discussing possible approaches to optimise patient management. The reader is also directed to a companion paper in this issue on indications for HSCT by Truong et al.

## Methods for MRD Measurement

MRD is the single most accurate predictor of event-free survival (EFS) in ALL. It is measured as the fraction of leukaemic cells in the bone marrow at pre-defined time points during the first months of therapy (9). Today, MRD is routinely measured by two sensitive methods: MFC and quantitative PCR (qPCR)-based analysis. Both techniques have strengths and pitfalls and there is a growing recognition of the need for both methods because they supplement each other in the management of B-cell precursor (BCP)-ALL and T-cell ALL, especially when aiming for correct stratification of virtually all patients (10–12). Droplet digital PCR (ddPCR) and next generation sequencing (NGS), especially, are promising technologies for MRD detection and are potentially useful as future methods for MRD monitoring, providing even higher sensitivity and accuracy than MFC and qPCR. However, standardisation of these methods is necessary before they can be applied in larger clinical series.

While ALL was previously considered a monoclonal disease, many studies have shown that—like other cancers—it may exhibit intra-tumoral heterogeneity, a common phenomenon in both BCP-ALL (13) and T-cell ALL (14). The clonal heterogeneity within individual patients may include therapy-resistant subclones which escape detection at the time of diagnosis (15, 16). Notably, heterogeneity is often, but not always, apparent when looking at the T-cell receptor (TCR) and B-cell receptor gene rearrangements; this has direct consequences for the sensitivity of MRD measurements based on Immunoglobulin H (IgH)/TCR markers (17–20). Correspondingly, intra-tumoral heterogeneity may be a challenge for defining stable and comprehensive leukaemia-associated immunophenotypes (LAIPs) useful for the measurement of MRD by MFC.

### PCR-Based MRD Analyses

The gold standard for MRD measurement in ALL is a qPCR-based method that uses clone/patient-specific PCR primers to amplify clonal rearrangements in IgH and TCR genes. These clonal Ig/TCR gene rearrangement sequences are detected by an initial Ig/TCR gene rearrangement analysis performed on the diagnostic sample—an analysis that has been developed through extensive work by the EU-founded consortia BIOMED-1 and BIOMED-2 (20).

**Figure 1:**
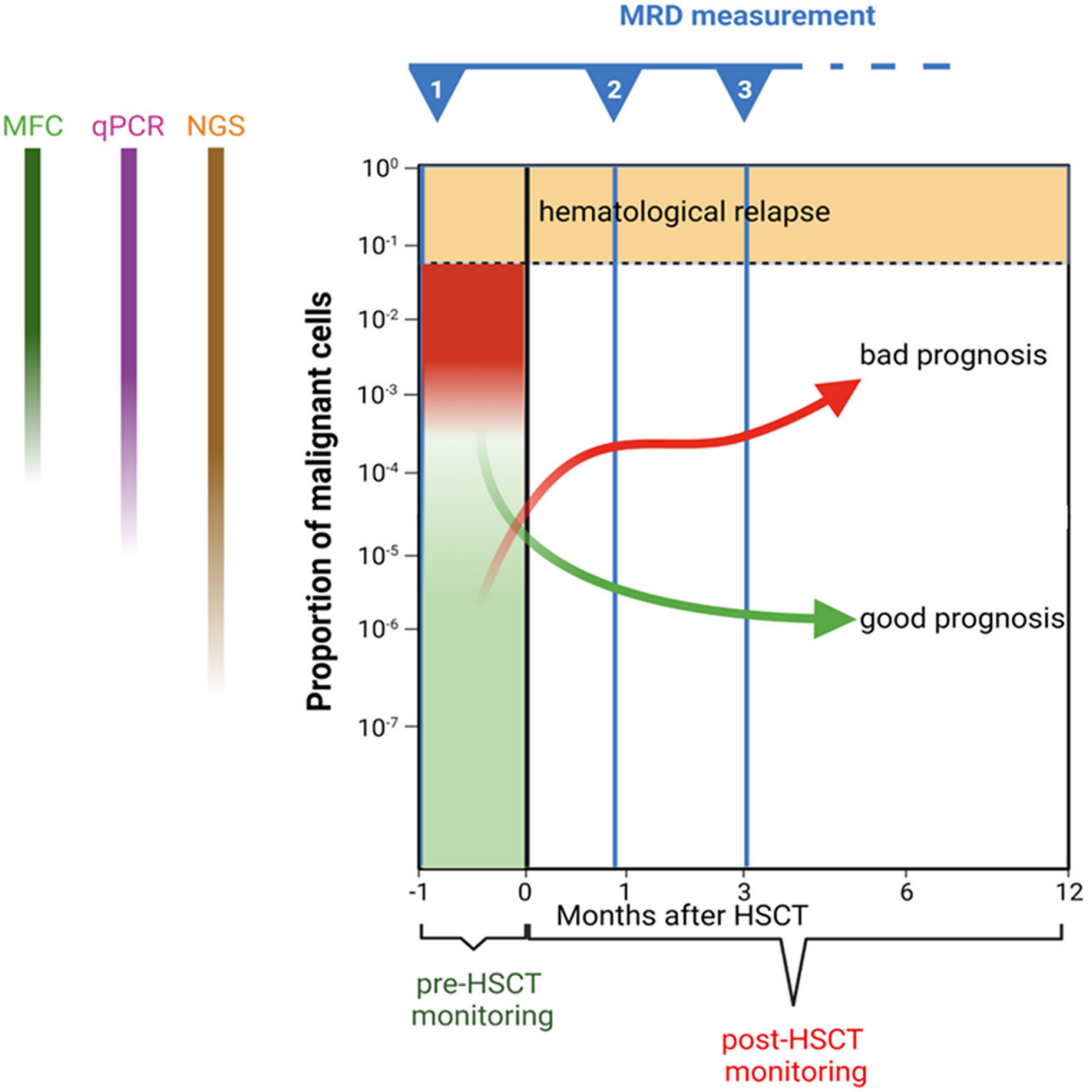
Schematic representation of minimal residual disease monitoring before and after haematopoietic stem cell transplantation. Trajectories of hypothetical patients with different prognoses are presented as examples.

qPCR is the longest-standing technique for measuring MRD and has been implemented for primary treatment stratification in most European childhood ALL protocols. Guidelines for set up and interpretation have been developed and implemented by the EuroMRD consortium (21, 22). The qPCR-MRD method is based on a standard dilution made from DNA of the diagnostic sample; for each patient-specific qPCR system, a limit for reproducible MRD results [named the “quantitative range” (QR)] and non-reproducible MRD results (the sensitivity of the analysis) are defined based on the EuroMRD criteria. The MRD level in follow-up samples is quantified by relating the qPCR signals to that of the standard dilution curve from the diagnostic sample. The method is highly reproducible and, in most cases, has a QR in the order of 10^−4^ and a sensitivity of 10^−5^ (see Figure 1). However, in 8–12% of ALL patients, no useful clonal IgH/TCR rearrangements are found (21); moreover, in those patients for whom useful markers are initially found, only 70 and 90% of the rearrangements are preserved following relapse of BCP-ALL and T-cell ALL, respectively (22, 23). A major problem of qPCR-based MRD detection is that the analysis targets dominating Ig/TCR rearrangements present in the bulk of the leukaemic population at the time of diagnosis and, hence, therapy-resistant subclones may remain undetected if they comprised only a small subset at diagnosis that was below the limit of detection of the Ig/TCR gene rearrangement analysis.

The same patient-specific PCR systems can be used in another PCR-based analysis: digital droplet PCR (ddPCR) (24). In ddPCR, through microfluidics and proprietary surfactant chemistries, the PCR sample is divided into thousands of water-in-oil droplets; thus, PCR amplification of the template occurs in each individual droplet; finally, the acquisition of data is performed at reaction end point. Thus, ddPCR has the advantage over qPCR that MRD can be measured without the involvement of a standard dilution curve. ddPCR seems to be more precise because of the nature of the technique, with the number of MRD-positive and -negative targets counted in each sample. Moreover, studies indicate that qPCR has a higher rate of false-positive MRD results in cases with non-quantifiable MRD than ddPCR (24). It is therefore likely that, in future, ddPCR will be the technique of choice over qPCR. However, the ddPCR technique needs standardisation before wide clinical implementation can take place.

### MFC-Based MRD Analyses

In MFC-based monitoring of MRD, leukaemic cells are distinguished from normal cells based on aberrant antigen expression, i.e., a LAIP (25). MFC-MRD is implemented and standardised as part of several international protocols for front-line ALL management [the Nordic Society of Paediatric Haematology and Oncology (NOPHO)-ALL2008, ALLTogether, and The Children's Oncology Group (COG) in North America] (6, 26, 27). MFC techniques have markedly improved: new fluorochromes for antibody conjugation have been developed leading to increased number of markers for investigation; moreover, MFC technologies (including hardware, software, and reagents) have been refined. Today, between 1 and 5 million cells in 8–10 colour/marker combinations are usually included in MFC-MRD, resulting in a lower limit of detection of at least 10^−4^ and a sensitivity of 10^−5^ in most cases depending on the number of cells in the sample, marker sensitivity (informative and stable LAIP), background signals (regeneration stage of bone marrow) and intra-tumoral heterogeneity.

MFC-MRD can be applied to most ALL patients (>90% of BCP-ALL cases have an informative LAIP), although it has been especially studied and implemented in BCP-ALL (11, 28, 29). MFC-MRD is also used successfully for treatment stratification in T-cell ALL (6, 10, 12, 30). In a NOPHO-ALL2008 study, it was shown that the MRD quantification by MFC was comparable to quantification by PCR and that MFC-MRD can be used in T-cell ALL in cases when no PCR-MRD marker is available. In MFC-MRD, the leukaemic cells are visualised directly and a result can be available within a few hours. In many cases, resistant subpopulations and marker modulation can be observed and followed (13). Another advantage of MFC-MRD is that the MRD value is calculated directly from the total number of cells in the sample; thus, increased sensitivity can be achieved by increasing the number of cells analysed, as employed in the recently initiated ALLTogether protocol. Noticeably, concordance between qPCR-MRD and MFC-MRD is directly correlated with the number of cells acquired; indeed, for those samples with >4 million cells, concordant results were obtained in 93% of samples (29). MFC-MRD is highly dependent on the presence of an immunophenotype (LAIP) that is informative (i.e., distinguishable from normal cells), a challenge that is particularly important during regeneration since haematogones resemble the blasts with regards to the majority of immunophenotypic markers investigated. Further, MFC-MRD is sensitive to marker modulation induced by particular therapeutic agents (i.e., downregulation or selection of mutated surface proteins following treatment with targeted immunotherapies) (31).

### Next-Generation Sequencing as a Future MRD Method

Contrary to traditional Sanger sequencing, NGS technologies are capable of sequencing multiple DNA molecules in parallel (known as “high throughput sequencing”) as well as generating sequence reads of a particular genomic region multiple times (also referred to as “deep sequencing”) (32). Thus, NGS technologies can potentially be used for quantifying MRD, i.e., by comparing the number of sequence reads with the number of reads from a known amount of reference DNA included in the sample (e.g., spiked-in DNA) (33). Alternatively, NGS technologies can be used to elucidate and track the entire repertoire of sequences within a particular genomic region, thus providing a unique possibility to visualise intra-tumoral heterogeneity and follow multiple leukaemic subclones.

The Ig/TCR amplicons are obvious candidates targets for NGS-MRD. However, a selection of genes routinely analysed for mutations in ongoing treatment protocols, including *NOTCH1, KMT2A*, and *IKAROS*, are also good candidates. Recently, the EuroClonality consortium reported on a standardised NGS method for target identification with IgH and TCR genes (34, 35); it holds great promise as a replacement to the existing multiplex PCR-based IgH/TCR gene rearrangement analysis. However, only a few studies have addressed the applicability of NGS for MRD purposes. A recent study points to NGS as a more sensitive method capable of demonstrating MRD in samples misclassified as MRD negative by the MFC-MRD method (see below) (36). Thus, NGS has the potential to be more sensitive than existing MRD methods. Theoretically, NGS-based approaches should allow for MRD detection at levels below 10^−5^, with some studies claiming sensitivities down to 10^−7^, i.e., far below that achievable with current qPCR-MRD or MFC-MRD (Figure 1). However, to achieve this level of sensitivity, a high amount of input DNA (i.e., many cells) is needed, which may prove difficult in hypocellular post-treatment samples (33).

Furthermore, NGS may allow the simultaneous monitoring of several leukaemic subclones within the same patient (i.e., account for intra-leukaemic heterogeneity) and thus provide an alternative means to detect residual disease in patients where the leukaemia undergoes immunophenotypic marker modulation and escapes detection by established MRD methods.

Research into the use of NGS for MRD detection is still in its infancy. Amongst the unresolved issues is a reliable standardised method for converting sequence reads to MRD levels, which must be developed before NGS-MRD can be put to diagnostic use (33). Also unknown are the sensitivity and the predictive power of NGS compared to known MRD techniques in larger patient cohorts. At present, the most immediate problem is to make NGS quantifiable for MRD purposes.

### MRD Measurement in Daily Practise

The method applied in HSCT centres may vary according to local practise and expertise. Currently, none of the available methods can be regarded as the sole gold standard; most centres use either qPCR or MFC but some may use both methods. For the purpose of assessing individual patients, it is important to identify the method that most likely reflects the true MRD level, taking possible subclones into consideration. Moreover, when interpreting results from studies, it is not always clear which level of MRD positivity was applied. Over the last 20 years, methods have been refined and so results from current studies may not be directly comparable with those from previous studies using less-sensitive techniques.

## Studies of MRD Assessment Prior to HSCT

**Figure 2:**
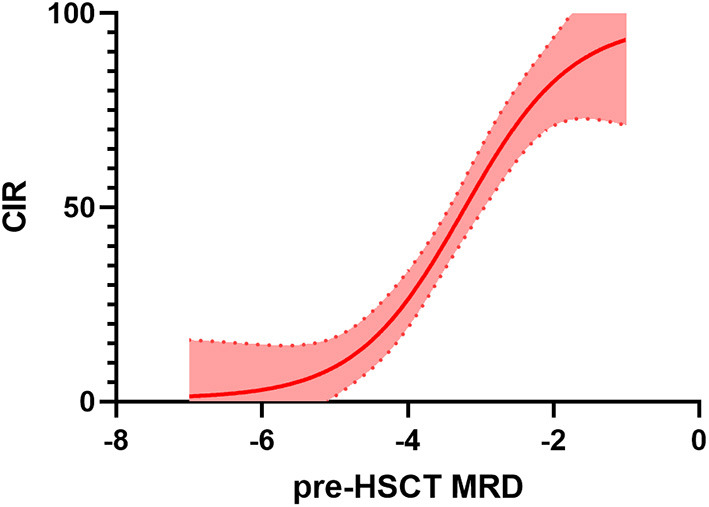
Relationship between minimal residual disease (MRD) before haematopoietic stem cell transplantation (HSCT) and cumulative incidence of relapse (CIR). Several studies (differing in population characteristics, method of MRD detection, transplant platform, etc., see also Table 1 for details) have been pooled together and interpolation has been performed. Thus, this can not be considered a methodologically solid analysis but an illustrative example of the increasing risk of relapse related to increases in pre-HSCT MRD.

Studies investigating MRD measurement prior to HSCT are outlined below and summarised in Table 1 (30, 36–48). In the late 1990s, Knechtli et al. in Bristol, UK, provided the first demonstration of the predictive role of PCR-MRD assessed prior to transplant in a retrospective analysis of 64 paediatric patients planned for allogeneic HSCT (37). In this first report, MRD was measured through semi-quantitative PCR (i.e., PCR products were size resolved by polyacrylamide gel electrophoresis and probed with labelled leukaemia-specific oligonucleotides) (49). The cumulative incidence of relapse (CIR) was 100% for patients with high-level pre-HSCT MRD (10^−2^-10^−3^), 45% for low-level MRD (10^−3^-10^−5^) and 20% for MRD-negative patients. Two-year EFS was 0, 36, and 73%, respectively (see Table 1 and Figure 2).

**Table 1:** Studies of MRD measurement prior to HSCT.

**References**	**Method**	**Patients, N**	**MRD subgroups**	**Outcomes**	**Notes**
Knechtli et al. (37)	PCR	64	Negative >10^−5^ to <10^−3^ >10^−3^ to <10^−2^	CIR: 20% 45% 100%	Semi-quantitative PCR
van der Velden et al. (38)	qPCR	17	Negative Positive	CIR: 20% 67%	First use of real-time qPCR
Bader et al. (39)	PCR	41	Negative Low-level positivity (>10^−4^ to <10^−3^) High-level positivity (>10^−3^)	EFS: 78% 48% 23%	Semi-quantitative PCR
Sramkova et al. (40)	qPCR	25	Negative Positive	LFS: 94% 0%	
Bader et al. (41)	qPCR	91	<10^−4^ ≥10^−4^	CIR: 13% 57%	
Leung et al. (42)	MFC	67^†^	<10^−4^ >10^−4^ to <5*10^−2^ ≥5*10^−2^	OS: 87% 48% 0%	
Pulsipher et al. (30)	MFC	105	Negative <10^−3^ ≥10^−3^	CIR: 25% 35% 60%	This was a randomised controlled trial evaluating the addition of sirolimus to standard GvHD prophylaxis in children with ALL.
Balduzzi et al. (43)	qPCR	82	<10^−4^ ≥10^−4^	CIR: 11% 61%	In the same study, post-HSCT MRD was evaluated (see Table 2).
Pulsipher et al. (36)	NGS	56	Negative Positive	CIR: 0% 16%	First study evaluating NGS-MRD in paediatric ALL. NGS-MRD predicted relapse and survival more accurately than MFC-MRD. In the same study, post-HSCT MRD was evaluated (see Table 2).
Sutton et al. (44)	qPCR	69	Negative Positive	LFS: 83% 41%	
Umeda et al. (45)	MFC	36	<10^−4^ ≥10^−4^	CIR: 27% 66%	
Lovisa et al. (46)	qPCR	119	Negative <10^−3^ ≥10^−3^	CIR: 20% 50% 73%	The level of MRD positivity had a different impact on EFS according to disease phase at HSCT (CR1 vs. CR2). In the same study, post-HSCT MRD was evaluated (see Table 2).
Ifversen et al. (47)	MFC and qPCR	66	<10^−4^ ≥10^−4^	CIR: 5% 23%	All patients were in CR1 following the same response-driven frontline protocol aiming to achieve pre-HSCT MRD <10^−3^.
Bader et al. (48)	MFC and qPCR	616	Negative <10^−4^ >10^−4^ to <10^−3^ >10^−3^	CIR: 20% 19% 35% 44%	Largest, multicentre study available. The combination of pre-HSCT and post-HSCT measurement increased the predictive value of the model.

† *Complete data were available for 33 ALL patients. ALL, acute lymphoblastic leukaemia; CIR, cumulative incidence of relapse; EFS, event-free survival; HSCT, haematopoietic stem cell transplantation; LFS, leukaemia-free survival; MFC, multiparametric flow cytometry; MRD, minimal residual disease; NGS, next-generation sequencing; OS, overall survival; PCR, polymerase chain reaction; qPCR, quantitative polymerase chain reaction*.

This first observation was later confirmed by Bader et al. using the same method in 41 children undergoing HSCT in first complete remission (CR1) or second complete remission (CR2) (39). Notably, as for the aforementioned study, multivariable analysis confirmed the independent prognostic significance of pre-HSCT MRD status.

The first study to evaluate qPCR for MRD measurement prior to HSCT (within a month before transplant) was conducted by van der Velden et al. in a small cohort of 17 paediatric patients who were classified as MRD negative or positive. The CIR was 20 and 67% for MRD negative and positive patients, respectively (38). Sramkova et al. reported similar results in a cohort of 25 patients with qPCR-MRD evaluable before HSCT. Remarkably, only one patient with positive (quantifiable) pre-HSCT MRD (about 10^−2^) did not relapse after HSCT; however, he died of infection 2 months after HSCT. Thus, in this small cohort, overall survival (OS) and leukaemia-free survival (LFS) of MRD-positive patients was 0% (40).

These initial findings were confirmed and further built upon through a trial conducted by the ALL-REZ Berlin-Frankfurt-Münster (BFM) Study Group in 91 children with relapsed ALL in whom MRD was assessed through qPCR (41). While the previous studies were performed retrospectively and included heterogeneous patient cohorts who received HSCT in different disease states (from CR1 to CR3) and who received different frontline and conditioning regimens, the ALL-REZ BFM study by Bader et al. was prospective and blinded. Among the 45 children with pre-HSCT PCR-MRD ≥10^−4^ the CIR was 57%, while in the 46 patients with PCR-MRD <10^−4^ the CIR was 13%. MRD proved to be the most important predictor for subsequent relapse and survival after transplantation in univariate and multivariate analysis.

MFC-MRD has been used by several study groups (e.g., COG, NOPHO, and ALLTogether), showing similar results in terms of prognostic value. In a cohort of 122 children with very-high-risk acute leukaemia, including 64 patients with ALL, Leung et al. showed that the 5-year cumulative incidence of relapse after HSCT was 6% in those with undetectable MRD, 16% among those with low levels of MRD (0.01 to <0.1% in ALL) and 40% in the patients with high levels of MRD (≥0.1% in ALL), as measured by MFC (42). Additionally, Pulsipher et al. investigated, in a COG/Paediatric Bone Marrow Transplant Consortium (PBMTC) multicentre Phase III trial, the addition of sirolimus to standard graft-*versus*-host disease (GvHD) prophylaxis in children with ALL, prospectively studying pre-HSCT MRD by MFC. Patients with MRD ≥0.1% had a higher CIR (60%) as compared to subjects who were MRD negative (25%) or had MRD <0.1% (35%) (30).

Balduzzi et al. reported their single-centre experience on the prognostic role of qPCR-MRD before and after allogeneic HSCT in 82 consecutive patients with ALL in CR (CR1–CR3) (43). They demonstrated that MRD status before transplantation had the strongest impact on outcome as compared to other prognostic factors, remaining highly relevant also after adjusting for post-transplant MRD pattern. Indeed, patients with qPCR-MRD <10^−4^ and ≥10^−4^ had a CIR of 11.4 and 61.5%, respectively. Noticeably, in contrast two other studies, the investigators were aware of the results of MRD testing; thus, they were able to rapidly intervene to reduce the risk of relapse. Indeed, of the 34 patients who had MRD levels ≥10^−4^ immediately before HSCT, 13 received treatment intensification with liposomal daunorubicin, fludarabine, and cytarabine (while the remaining 21 proceeded directly to HSCT). Eight out of 13 responded to intensification, with MRD levels reduced below 10^−4^; all eight were in CR after HSCT without further interventions, while the three out of five patients who did not respond to intensification relapsed after HSCT.

Lovisa et al. retrospectively studied the impact of pre-transplant and post-transplant (see below) qPCR-MRD in 119 consecutive patients aged between 1 and 18 years affected by ALL in CR1, CR2 or subsequent morphological CR given allogeneic HSCT using one of the Associazione Italiana di Ematologia e Oncologia Pediatrica (AIEOP) protocols (46). Details are reported in Table 1. One of the main findings was that pre-HSCT MRD had a different impact on outcome based on the disease status of the patient. Indeed, for patients transplanted in CR1, the EFS probability was similar if pre-HSCT MRD was negative or low (i.e., <10^−3^), while for patients transplanted in CR2, any MRD positivity was associated with a poor prognosis. Furthermore, the authors showed a strong correlation between disease phase and pre-transplant MRD level; in fact, pre-transplant MRD negativity was observed more frequently in patients transplanted in CR1 and in those transplanted in CR2 and belonging to the BFM S1–S2 risk groups. Also in this study, clinically significant (i.e., grade II–IV) acute GvHD demonstrated a protective effect against relapse, especially in patients with pre-transplant low-level MRD positivity.

Recently, different cooperative groups from Europe, North America, and Australia (i.e., the COG, PBMTC, Australian Transplantation Group, International BFM Study Group, Paediatric Diseases Working Party of the European Society for Blood and Marrow Transplantation, and all members of the Westhafen Intercontinental Group) created an international database of 616 paediatric patients to allow a more precise and detailed statistical analysis of the predictive power of MRD in the context of other independent risk factors through risk modelling (48). Moreover, the database gave insight on: (1) the relationship between different methods of MRD quantification; (2) when in the course of the HSCT process MRD measures matter the most; (3) what are the implications of serial positivity of MRD; and (4) what clinical factors post HSCT can modify the course of patients who are MRD positive either before or after the HSCT. This analysis included two standardised approaches for MRD measurement, namely the EuroMRD qPCR approach used in Europe and Australia and the COG MFC method used in North America.

In line with previous studies, the collaboration found that detectable pre- and post-HSCT MRD was strongly associated with both relapse and EFS, with higher MRD predicting higher CIR and inferior EFS (Table 1). Notably, the authors analysed the relative impact of pre- and post-transplant MRD positivity on outcome; this was assessed through bivariate analysis and by computing the proportion of explainable log-likelihood by each variable minus its degree of freedom. They found that pre-HSCT MRD positivity was less important than post-HSCT positivity, accounting for 7% and 57% of explainable risk of relapse, respectively. Compared to MFC, qPCR showed increased sensitivity; however, because HSCT outcomes for patients with lower-level MRD vs. undetectable MRD did not differ, it remains to be clarified the clinical relevance of undetected low-level MRD in the MFC cohort; furthermore, the patients were analysed by one method only, thus direct comparison between the methods was not possible. Additionally, the authors used a multivariable Fine-Grey regression model to assess the impact of risk factors on relapse; they found that besides MRD positivity before HSCT, other independent pre-HSCT risk factors for relapse were disease status (i.e., CR2 or ≥CR3 remission status) and use of non-TBI-based conditioning regimens. Thus, combining these factors they created and validated a risk score model able to classify patients in three groups, with good, intermediate, and poor prognosis.

Several groups have shown the value of NGS technologies for MRD detection (36, 50). When NGS-MRD was compared with MFC-MRD in 56 paediatric patients with B-cell ALL enrolled in the ASCT0431 COG study, NGS-MRD predicted relapse and survival more accurately than did MFC-MRD. Indeed, the 2-year relapse probabilities were 53 and 0% among NGS-MRD positive and negative patients, compared with 46 and 16% among MFC-MRD positive and negative patients, respectively (36). Despite being obtained in a relatively small cohort, the finding that patients with pre-transplant negative NGS-MRD did not relapse is particularly interesting, indicating that increasing the sensitivity of detection may identify patients at low/very-low risk of relapse. The PBTMC EndRAD trial is currently studying whether patients with negative NGS-MRD before HSCT can be treated with a radiation-free conditioning protocol (Clinicaltrials.gov identifier: NCT03509961). Moreover, it has to be noted that, in patients with positive pre-HSCT NGS-MRD, there was no trend in relapse rates by quantity of residual leukaemia, with relapse occurring frequently even at the lowest levels of detection (i.e., 10^−6^). Finally, as subsequently confirmed by other authors, clinically significant acute GvHD was associated with a reduced incidence of relapse in patients with positive pre-HSCT NGS-MRD.

The NOPHO cooperative group reported the impact of pre-HSCT MRD for patients treated with the ALL2008 protocol (47). Notably, all patients were in CR1 at time of transplant and were homogeneously treated according to the same protocol (in which risk stratification was based only on MRD). They confirmed that patients with negative MRD prior to transplantation had a very low risk of relapse (i.e., 5.1%). In the multivariable analysis, MRD positivity ≥10^−4^ was the only variable significantly associated with relapse, with a hazard ratio of 9.1.

In the case of other genetic markers being available for qPCR-MRD monitoring (e.g., *BCR-ABL1*), it is not clear whether MRD measurement using these would be more informative than using MFC or Ig/TCR qPCR. In a childhood ALL study, Cazzaniga and co-authors evaluated both *BCR-ABL1* qPCR and Ig/TCR qPCR at different time points; concordance between the two methods was only 69%, with Ig/TCR-based MRD levels appearing the more reliable predictor of outcome following standard therapy consisting of chemotherapy and Imatinib (51). However, similar data are not available in the HSCT setting.

To summarise, from these different studies it can be argued that the lower the level of pre-HSCT MRD, the lower the risk of relapse and, finally, the better the outcome. However, it is still unclear: (1) whether levels of MRD analysed by qPCR and MFC are interchangeable; and (2) what is the best approach to treatment in case of MRD positivity (see also below).

## Studies of MRD Assessment After HSCT

From the data available, it is clear that MRD assessment before transplantation cannot effectively identify all individuals with impending post-transplantation relapse who might benefit from pre-emptive intervention. For this reason, the predictive role of post-transplant MRD has been investigated by several groups (36, 43, 46, 48, 52, 53) (Table 2).

**Table 2:** Studies of MRD measurement after HSCT.

**References**	**Method**	**Timing after HSCT**	**Patients, N**	**MRD subgroups**	**Outcomes**	**Notes**
Balduzzi et al. (43)	qPCR	Days +30, +60, +90, +180, +270, +365	82	Positive (any value, any time) ≥10^−4^ to <10^−3^ (any time) ≥10^−3^ (any time)	EFS: 40% 28% 0%	All patients who experienced >1 log increase in MRD after transplant ultimately relapsed.
Bader et al. (52)	qPCR	Days +30, +60, +90, +180, +365	113	Negative <10^−4^ ≥10^−4^	CIR (day +60): 23% 42% 75%	The accuracy of MRD measurements for predicting relapse was investigated with time-dependent receiver operating curves at days +30, +60, +90, and +180. From day +60 onward, the discriminatory power of MRD detection to predict the probability of relapse after 1, 3, 6 and 9 months was >96, >87, >71, and >61%, respectively.
Pulsipher et al. (36)	NGS	Days +30, +100, +240, +360	53	Negative Positive	CIR: 13% 73%	Relapses in NGS-MRD negative patients may reflect incomplete sampling of hypoplastic marrow; indeed, NGS-MRD libraries prepared at the 30-day time point contained significantly fewer total sequences than those prepared at any other time point.
Lovisa et al. (46)	qPCR	Day +30 Day +90	98 59	Negative <10^−3^ ≥10^−3^ Negative <10^−3^ ≥10^−3^	EFS: 63% 30% 25% EFS: 84% 44% 0%	The “kinetics” of MRD (i.e., increase or decrease between the different time points) influenced outcome.
Bader et al. (48)	MFC and qPCR	Days +30, +60, +90, +180, +360	Median 353 (range 218–386)	Negative <10^−4^ ≥10^−4^ to <10^−3^ ≥10^−3^	HR (vs. MRD negative): 1 1.65 4.39 14.58	A Cox regression model, which considered MRD levels pre HSCT in the context of post-HSCT MRD assessments, showed that patients with high and very-high pre-HSCT MRD positivity who obtained post-HSCT MRD negativity had low CIR and high EFS.

In a seminal BFM study of 113 paediatric patients transplanted for relapsed ALL, the level of PCR-MRD was inversely correlated with EFS and positively correlated with CIR at all time points after transplant. In a multivariable analysis, an MRD ≥10^−4^ was consistently correlated with inferior EFS (52). Although high levels of post-transplant MRD were strongly predictive of disease recurrence, low-level MRD positivity after transplantation was not invariably associated with relapse, especially if detected early after HSCT. However, this and several other studies have shown that the greater the time that has lapsed since HSCT was performed, the more likely that even low levels of MRD will predict poor prognosis (43, 46, 52, 53). Indeed, in the study by Balduzzi et al. (43), MRD positivity after transplantation was associated with a 2.5-fold higher risk of treatment failure when detected early (in the first 100 days after HSCT) yet a 7.8-fold higher risk when detected subsequently (i.e., at 6, 9 or 12 months post HSCT). However, it has to be noted that qPCR-MRD levels <10^−4^ (i.e., those defined as “positive not quantifiable” at best of technical requirements according to EuroMRD rules) may represent “false positives,” due to unspecific binding of patient-specific primers at the time of intense B-cell regeneration (54). These findings support the assumption that low levels of residual leukaemia cells could be controlled by an immunologic graft-*versus*-leukaemia effect in the early post-transplant period before the graft becomes tolerant toward the recipient.

In the aforementioned study by Lovisa and co-authors (46), patients with positive MRD <10^−3^ or ≥10^−3^ 1 month after HSCT had an EFS probability of 30 and 25%, respectively; for the same levels at 3 months after HSCT, the EFS probability was 44 and 0%, respectively. Moreover, this study confirmed the data by Bader et al. (52) showing that MRD evaluation is a dynamic process and that variations of MRD over time are important (Figure 1). This concept was further supported by the Westhafen Intercontinental Group study led by Bader et al. (48). As already outlined above, post-HSCT positivity had a high prognostic value, accounting for more than 50% of the risk of relapse. Indeed, the authors underlined that although high-risk patients could be identified before HSCT, a significant percentage of relapses occurred in patients who had low MRD positivity or were MRD negative prior to HSCT, once more indicating that these relapses might be identified early by frequent post-HSCT MRD monitoring. Additionally, they defined very-high-risk groups that may benefit from more frequent MRD assessment (e.g., those patients with MRD positivity before transplantation, those in CR ≥2, those not receiving TBI in the conditioning regimen, and those not developing acute GvHD by day +90). Indeed, in that study, which had sufficient statistical power to analyse several risk factors for relapse, both MRD negative and positive patients had an approximately 3-fold decrease in relapse risk if they developed acute GvHD. Patients who had positive MRD after HSCT and developed acute GvHD had relapse rates similar to those who were MRD negative and did not develop aGvHD.

The beneficial effect of acute GvHD on relapse risk and survival of children with ALL has been documented by other reports. In a COG/PBMTC study, patients with pre-HSCT MFC-MRD ≥0.1% who did not develop acute GvHD compared with those with MFC-MRD <0.1% who developed acute GvHD had much worse 2-year disease-free survival (DFS) (18 vs. 71%, respectively). Patients with pre-HSCT MRD <0.1% who did not experience acute GvHD had higher rates of relapse than did those who developed acute GvHD (40 vs. 13%, respectively) (53). In patients with B-cell ALL, post-HSCT MRD positivity detected by NGS was more predictive of relapse than that detected by MFC, especially early after HSCT: at day +30, the relapse rate was 67 vs. 35% in NGS-MRD positive patients vs. MFC-MRD positive patients, respectively, and 25 vs. 30% for NGS-MRD negative patients vs. MFC-MRD negative patients. Any post-HSCT NGS-MRD positivity resulted in an increase in relapse risk in the multivariate analysis (HR 7.7) (36). The improved predictive ability of NGS-MRD was primarily attributed to the higher sensitivity of this methodology. Among 11 patients who were NGS-MRD positive but MFC-MRD negative post HSCT, seven relapsed. On the contrary, none of the patients positive by MFC-MRD but negative by NGS-MRD relapsed.

In summary, available data suggest that: (1) post-transplant MRD positivity is not invariably associated with relapse and can be modified by the presence of acute GvHD; (2) as expected, higher post-HSCT MRD levels are associated with higher risk of relapse (up to 80–100% for MRD >10^−3^); (3) the later the MRD positivity occurrence, the higher the risk of relapse; (4) serial and tight monitoring of post-HSCT MRD is more predictive of relapse risk compared to pre-HSCT positivity and can guide risk-adapted intervention as well as the evaluation of response to such therapies (see also below); and (5) NGS-MRD analyses both pre and post HSCT might provide a more sensitive tool to predict relapse, but current data need further confirmation and validation in additional cohorts.

## Other Techniques to Evaluate Residual Disease

### ^18^F-FDG-PET/CT

^18^F-fluorodeoxyglucose positron emission tomography/computed tomography (^18^F-FDG-PET/CT) is an established tool for the diagnosis and follow-up of lymphoma. For the initial diagnosis of leukaemia, it is not used as information from blood and bone marrow is sufficient to establish the disease status. At time of imminent or overt relapse, ^18^F-FDG-PET/CT can contribute to the discovery of focal disease, sometimes early on, providing subsidiary information that standard MRD quantitation might not reveal.

Zhao et al. retrospectively analysed findings from ^18^F-FDG-PET/CT performed before and/or after HSCT for acute leukaemia in 72 patients (55). The study included various types of leukaemia and evaluated bone marrow, lymph nodes, spleen and extramedullary disease. Notably, extramedullary disease as detected by ^18^F-FDG-PET/CT was significantly associated with disease status and OS, especially when assessed post transplantation. While extramedullary disease is considered a more frequent event in acute myeloid leukaemia (AML) than in ALL, its impact on prognosis is being debated (56–58). For ALL, extramedullary disease is systemically monitored by assessing central nervous system (CNS) disease and testicular involvement, whereas lesions in the bone may only be detected when causing symptoms. Furthermore, localised relapse in the bone or bone marrow has been observed in patients without systemic involvement (59–61).

To properly evaluate the specificity of ^18^F-FDG-PET/CT, analysis will need to focus on different organs separately because infection, inflammation (e.g., due to GvHD), and haematological regeneration may have an impact on measurements. These data on specificity are needed to avoid unnecessary follow-up exams (such as biopsies) due to false-positive results. Moreover, it has to be considered that, globally, the sensitivity of ^18^F-FDG-PET/CT is lower than that of MFC or PCR. However, especially in the relapse setting and in the context of HSCT and immunotherapy, one may not necessarily rely on the assumption that bone marrow assessment alone is sufficient to track focal disease.

In conclusion, ^18^F-FDG-PET/CT is not needed at diagnosis of ALL and current data are insufficient for a general recommendation to use ^18^F-FDG-PET/CT in the relapsed-disease setting as an additional diagnostic tool. In singular cases, and at specific time points (e.g., prior to HSCT), it may add valuable information.

### Chimerism

In the post-transplant setting, although less sensitive than MFC-MRD and qPCR-MRD, close chimerism monitoring of peripheral blood has proven useful for the early detection of impending relapse in ALL (62, 63). In seminal work by Bader and co-authors, serial analysis of chimerism by fluorescent-based short-tandem-repeat PCR was performed in 163 children with ALL undergoing HSCT. Patients were classified as having complete chimerism/low-level mixed chimerism (*n* = 101), increasing mixed chimerism (*n* = 46), or decreasing mixed chimerism (m = 16). The highest incidence of relapse was found in patients with increasing mixed chimerism, with 26 out of 46 patients experiencing disease recurrence. Notably, no relapse was reported in the decreasing mixed chimerism group, highlighting once more the importance of serial evaluations and dynamic risk stratification (63).

Chimerism analysis can be coupled with other techniques to increase the sensitivity and specificity of the method. Semchenkova et al. isolated by flow cell sorting questionable cell populations identified with MFC-MRD monitoring and analysed them for chimerism by qPCR (64). The analysis was successful in 50 out of 52 patients in whom low-level MRD positivity was suspected; in 62% of cases the analysis confirmed the recipient origin of the cells, while in the remaining 38% of cases all cells were of donor origin, thus excluding MRD relapse.

Using qPCR, increased sensitivity has been achieved, allowing for earlier detection of impending relapse in adult (65) as well as in paediatric series (66). In both studies repeated measurement of increasing mixed chimerism in peripheral blood was significantly correlated with relapse, thus adding to the number of tools for assessment of relapse risk. The method is yet not validated in larger series, but may be a promising tool to spare selected patients from MRD-assessment in bone marrow in general anaesthesia (e.g., patients who are MRD-negative at day 30 or 60).

## Data From Recent Clinical Trials

In the international, multicentre, prospective, Phase 3 FORUM study, the question of MRD was prospectively evaluated as a risk factor for outcome. Pre-HSCT MRD was assessed at a maximum of 14 days prior to start of conditioning. The protocol suggested, but did not mandate, that MRD was tested post HSCT at day +30, +60, and +100 as well as at 6, 9, 12, 15, and 18 months. MRD was defined as positive if MFC-MRD was >10^−3^ or PCR-MRD was >10^−4^, both analyses due to be performed in laboratories participating in the European Scientific Foundation of Laboratory Hemato-Oncology. In the published cohort, only pre-HSCT MRD was analysed as a risk factor, and with data completeness of 81%. Pre-HSCT MRD was positive per study definitions in 144 patients (132 by PCR and 12 by MFC), whereas 192 patients were MRD negative, thus the positive fraction comprised 42% (67). Surprisingly, positive pre-HSCT MRD did not influence either OS or EFS in the multivariable analysis.

In previous studies, MRD was associated with relapse or EFS, even with fewer patients at risk. Indeed, in the already-reported prospective COG study, patients with MFC-MRD ≥10^−3^ had a 3-fold risk of relapse as compared to that of MRD-negative patients (30). In the retrospective NOPHO study including patients in CR1 only, 22 of 69 patients (32%) were MRD positive pre HSCT and these patients had an increased risk of relapse as compared to MRD-negative patients (47). Furthermore, the seminal study from the Westhafen Intercontinental Group clearly showed a negative impact of MRD on relapse and EFS in the validation cohort, with EFS of 71% in the MRD-negative/very-low group vs. 58 and 37% in the MRD-high and MRD-very-high groups, respectively (48).

The reason for pre-HSCT MRD not being significantly associated with EFS in the FORUM study is not clear. The inclusion of patients into the study required patients being in CR, without limitations on MRD levels, yet most upfront or relapse protocols aimed to induce low level of MRD (i.e., <10^−3^ pre HSCT). It is likely that new drugs and new approaches may have induced better leukaemia control despite MRD positivity immediately prior to HSCT. Whether post-HSCT MRD levels at day +60 or +100 combined with the presence of controlled acute GvHD was predictive of outcome in the FORUM cohort will be analysed separately. Furthermore, analysis of the precise levels of MRD pre HSCT may further elucidate whether low levels of MRD contributed to the fact that MRD did not influence the cohort.

## MRD-Guided Interventions

The evaluation of MRD post HSCT may identify patient at high risk of relapse and provide an opportunity to intervene using several different approaches. Overall, these approaches attempt to gain control over any residual leukaemia by: (1) inducing a graft-*versus*-leukaemia effect; or (2) directly targeting the residual leukaemia cells.

Historically, the first approach to reduce the risk of relapse in patients with detectable residual disease or decreasing donor chimerism was the rapid withdrawal of immune suppression. The development of GvHD during the withdrawal of immune suppression was cautiously regarded as a “success” in the hope of inducing a graft-*versus*-leukaemia effect. With the use of MRD surveillance post HSCT, this approach remains a reasonable practise yet there is controversy over when to intervene (e.g., at what level of detectable MRD) and how quickly to withdraw immune suppression.

If withdrawal of immune suppression is not successful or the patient has already ceased immune suppression, then donor lymphocyte infusions (DLI) are an alternative method to induce a graft-*versus*-leukaemia effect. In a multicentre French study by Pochon et al., 133 children with ALL who underwent myeloablative conditioning and HSCT had PCR-MRD surveillance at days −30, +30, +90, and +150 of transplant (68). Patients who had MRD ≥10^−3^ at any time point had rapid withdrawal of cyclosporine and those who did not respond proceeded to receive DLI. Interestingly, the group found that withdrawal of cyclosporine resulted in the clearing of MRD but, ultimately, reducing the duration of cyclosporine in MRD-positive patients did not prevent relapse. When comparing their data with that of Balduzzi et al. (43), similar rates of acute GVHD were found regardless of pre-emptive immune intervention. Importantly, very few patients (*n* = 9) received pre-emptive DLI, emphasising that this is not a feasible approach due to early haematological relapse or poor patient status.

A recent study by Rettinger et al. used both chimerism and post-HSCT MRD measurement to guide pre-emptive immunotherapy (i.e., discontinuation or tapering of immunosuppressive therapy for patients still receiving it in the early post-transplantation period or administration of DLI as frontline therapy in patients not receiving immunosuppressive therapy) (62). Nine patients discontinued immunosuppressive therapy (at a median of 45 days after transplantation), 11 received DLI (at a median of 150 days after HSCT), and three underwent both discontinuation of immunosuppressive therapy and administration of DLI. Interventions did not result in an increased risk of GvHD; notably, CIR and TRM in the intervention groups were similar to those of 66 patients who did not receive any intervention because of complete chimerism and/or negative post-HSCT MRD. There was no difference in outcome between patients who ceased immunosuppressive therapy and those who received DLI.

Recent approaches to address MRD positivity employ immunotherapy to directly target residual leukaemia in the post-HSCT setting. Blinatumomab is a bi-specific T-cell engager antibody that has dual specificity for CD19 and CD3, bringing T cells in close proximity to CD19-positive ALL cells thus facilitating cytotoxic tumour cell killing. In both paediatric and adult studies on relapsed/refractory BCP-ALL, blinatumomab induced rapid and high responses even in heavily pre-treated patients and patients with relapses post HSCT (see also the companion paper in this supplement by Krauss et al.) (69–76).

In the post-transplant setting, several collaborative group studies are underway to evaluate the use of blinatumomab in patients who are MRD positive. The ongoing FORUM study was amended to introduce a limited-institution sub-study to evaluate the use of blinatumomab in patients with positive MRD post HSCT (Clinicaltrials.gov identifier: NCT04785547). The primary endpoint is the rate of MRD negativity (defined as <0.01% by MFC or <10^−4^ by PCR) after one or two blinatumomab cycles post HSCT. Patients with positive MRD pre HSCT are eligible for this add-on study and will receive blinatumomab between day +60 and day +100 post HSCT, while patients who become MRD positive post HSCT receive blinatumomab between day +60 and +360 post HSCT. Similarly, the Canadian Transplant and Cellular Therapy Group is also evaluating in a prospective fashion the use of blinatumomab for patients with BCP- ALL who are MRD positive post HSCT (Clinicaltrials.gov identifier: NCT04044560).

Other pre-emptive approaches involve using antibody–drug conjugates that target residual leukaemia cells such as inotuzumab ozogamicin (an anti-CD22 antibody linked to calicheamicin) or moxetumomab pasudotox (an anti-CD22 antibody linked to a *Pseudomonas* exotoxin). These agents may be used to sustain remission or as a bridge to a second transplant. Finally chimeric antigen receptor (CAR) T-cell therapy has been reserved for patients with MRD positivity who develop full blown relapse.

CAR T-cell therapy might be an option for a carefully selected subgroup of BCP-ALL patients with MRD positivity either pre or post HSCT (see also the companion paper in this issue by Buechner et al.). Tisagenlecleucel, the only commercially available CD19-directed CAR-T cell therapy for paediatric patients with BCP-ALL, is approved by the US Food and Drug Administration and European Medicines Agency for the indication of a second or higher relapse, a relapse post HSCT, or refractory disease at primary diagnosis or relapse. Thus, depending on national regulations and reimbursement policies, a patient with persistent MRD much beyond the level of what is acceptable prior to HSCT despite therapy intensification efforts might in some centres be classified as “refractory” (although having <5% bone marrow blasts) and be a candidate for CAR-T cell therapy as potential standalone therapy rather than blinatumomab as a bridge to transplant. However, such strategies should implement careful documentation and evaluation by real-world CAR T-cell registries to capture data on outcomes, patients' overall treatment journeys, and costs.

The only active study prospectively evaluating tisagenlecleucel in an MRD-positive setting is the multicentre Phase II CASSIOPEIA trial (Clinicaltrials.gov identifier: NCT03876769). The trial is enrolling patients 1–25 years of age with *de novo* National Institutes for Health-defined high-risk BCP-ALL who are MRD positive (≥0.01% by MFC) at the end of consolidation. Such high-risk patients would, if not enrolled into CASSIOPEIA, be stratified to HSCT in CR1 by most front-line protocols for ALL management. In CASSIOPEIA, however, patients will not undergo HSCT if they remain in MRD-negative remission after CAR T-cell therapy, with the option of a CART-cell re-infusion in the case of MRD reappearance or early B-cell recovery.

Lastly, as discussed above, MRD positivity post HSCT—especially at later time points and at higher levels—is a strong predictor of subsequent relapse. Therefore, in a patient with a clearly rising MRD >5–6 months post HSCT who is not taking immunosuppression and does not have signs of GvHD, centres who have access to tisagenlecleucel (or other investigational agents) and do not participate in blinatumomab intervention studies might decide to proceed to CAR T-cell therapy although the patient has <5% blasts in the bone marrow. Considerations behind the decision could be that the rising MRD will inevitably progress to frank relapse, and—as CAR-T cell therapy is more effective in patients with a lower rather than high blast count (77–79)—the earlier CAR T-cell manufacturing is initiated, the more likely it is that the patient will not need bridging chemotherapy prior to CAR T-cell infusion.

In conclusion, some patients with BCP-ALL remaining MRD positive during front-line therapy or relapse therapy might be allocated to tisagenlecleucel or other CAR T-cell products after thorough considerations to either prevent a frank relapse or to avoid HSCT. However, such individualised interventions should preferably be done in the context of controlled studies and/or, as they are off-label, be thoroughly documented in CAR T-cell therapy registries to understand their impact on outcomes and toxicities. The established path to tisagenlecleucel post HSCT is when an MRD positive patient progresses to full blown relapse. Notably and similarly to the post-HSCT setting, NGS-MRD post CAR T-cell infusion was more sensitive than MFC-MRD to detect impending relapses: in a relapsed/refectory BCP-ALL cohort, NGS-MRD negativity at day 28 post infusion predicted a superior 3-year relapse-free survival of 80% compared to 20% in patients who were NGS-MRD positive at any level (80).

## Conclusions

As clearly demonstrated by several studies, the best pre-transplant status in terms of prognosis is MRD negativity. This is regardless of the technique used, with more sensitive methods (i.e., NGS) predicting the best results. Increasing the sensitivity of the technique used (up to 10^−7^ with NGS) increases our ability to predict the risk of relapse of a given patient, thus further optimising patient management.

While MFC and qPCR are now highly standardised and reproducible between different laboratories, NGS still needs inter-centre standardisation for the different phases of testing (including use of control quantification material); moreover, quality assessment and informatics analysis of high throughput sequencing data are still lacking, which is being addressed by the EuroClonality NGS Consortium. Noticeably, in the study by Pulsipher et al. on NGS-MRD monitoring pre and post HSCT, five out of 38 patients with constantly negative NGS-MRD relapsed (36). This may be due to incomplete sampling of a hypoplastic marrow; indeed, NGS-MRD libraries prepared at the 30-day time point contained significantly fewer total sequences than any other time point, reflecting the characteristic lymphopenia of this post-transplant period.

One important limit of all studies on MRD performed in the HSCT setting is that, because of the relatively low numbers of patients enrolled, MRD has been analysed as a dichotomous variable instead of a continuous one, thus leading to loss of statistical power and reduction of predictive accuracy (81). Indeed, in non-transplant studies, recent evidence suggests that analysing MRD as a continuous variable and integrating different risk factors allows more refined risk stratification (82, 83).

For patients with pre-HSCT MRD positivity, it is still not clear what is the best treatment strategy. Indeed, clearance of MRD is desirable but pre-transplant therapy intensification poses risks of complications, delay to HSCT and, ultimately, loss of the window for HSCT if disease progression occurs (84, 85). Notably, in the NOPHO ALL2008 protocol, longer time between diagnosis and transplantation was associated with increased TRM, possibly reflecting the fact that additional treatment courses aimed at decreasing MRD prior to HSCT resulted in higher toxicity (47). Conversely, in the study by Balduzzi and co-authors, treatment intensification before HSCT aimed at reducing MRD <10^−4^ was associated with a 5-fold reduction in the hazard of death (43).

The recent publication of two randomised controlled trials on the use of blinatumomab in first relapse of ALL conducted by the COG (86) and IntReALL Consortium (87) showed that the bispecific T-cell engager was superior to conventional chemotherapy to prepare children to HSCT. Indeed, the two independent trials showed that: (1) regardless of the timing of randomisation or type of chemotherapy given, blinatumomab was significantly less toxic than chemotherapy (infections and sepsis, frequently responsible for delay in proceeding to HSCT, were less common with blinatumomab); (2) blinatumomab use was associated with higher rates of MRD negativity than the chemotherapy-only groups; (3) patients who received blinatumomab as consolidation chemotherapy were more likely to proceed to HSCT than patients receiving standard chemotherapy; and (4) these differences translated into superior DFS and OS for children treated with blinatumomab prior to HSCT. Thus, the current possibilities with seemingly less toxic pre-HSCT therapies in case of relapsed or refractory disease or a post-HSCT rise in MRD may alter the dynamics of post-HSCT morbidity, TRM and relapse risk.

Finally, studying the scenario of post-HSCT MRD positivity in patients who do not relapse is probably even more interesting than investigating mechanisms of relapse in pre-HSCT MRD negative patients. Indeed, in-depth study of this group of patients may help us to better design effective (and less risky) pre-emptive treatment strategies.

## Author Contributions

All authors drafted the article, reviewed and revised the manuscript, and approved the final version of the manuscript.

## Conflict of Interest

JB has received personal fees, advisory board/steering committee honoraria, and nonfinancial support from Novartis; and advisory board honoraria from Pfizer, Kite, and Janssen. The remaining authors declare that the research was conducted in the absence of any commercial or financial relationships that could be construed as a potential conflict of interest.

## Publisher's Note

All claims expressed in this article are solely those of the authors and do not necessarily represent those of their affiliated organizations, or those of the publisher, the editors and the reviewers. Any product that may be evaluated in this article, or claim that may be made by its manufacturer, is not guaranteed or endorsed by the publisher.
